# Associação entre violência na comunidade e o risco de insegurança alimentar em uma capital do Sul do Brasil

**DOI:** 10.1590/0102-311XPT034424

**Published:** 2024-11-22

**Authors:** Francielle Veloso Pinto Pereira, Raquel Canuto, Ilaine Schuch

**Affiliations:** 1 Universidade Federal do Rio Grande do Sul, Porto Alegre, Brasil.

**Keywords:** Insegurança Alimentar, Ambiente Social, Características da Vizinhança, Violência, Food Insecurity, Social Environment, Neighborhood Characteristics, Violence, Inseguridad Alimentaria, Ambiente Social, Características del Vecindario, Violencia

## Abstract

A insegurança alimentar e a violência urbana estão entre os principais problemas de saúde pública no Brasil, possuindo aproximadamente metade da população com prejuízo no acesso a alimentos, além de grande parte dos indivíduos já terem experienciado alguma situação de violência na vizinhança em que vivem. Estudos têm demonstrado que a violência na vizinhança pode estar associada à insegurança alimentar; entretanto, no Brasil, essa temática é pouco explorada. O objetivo deste estudo foi verificar a associação entre a percepção de violência na vizinhança e o risco de insegurança alimentar. Este estudo tem desenho transversal, realizado com adultos e idosos (n = 400) residentes em uma área de saúde da cidade de Porto Alegre, Rio Grande do Sul. Foi adotado um método de amostragem estratificada. A coleta de dados ocorreu por meio de entrevistas domiciliares. A presença de risco de insegurança alimentar e a percepção da violência na comunidade foram avaliadas por meio de instrumentos validados para a população brasileira. Regressão de Poisson com variância robusta foi utilizada para estimar a razão de prevalência (RP) brutas e ajustadas e seus respectivos intervalos de 95% de confiança (IC95%). Indivíduos com percepção que a sua vizinhança era violenta, apresentaram maior probabilidade de apresentar risco de insegurança alimentar (RP = 1,35; IC95%: 1,04-1,77). A percepção de violência na vizinhança está associada ao risco de insegurança alimentar de forma independente, após ajuste para possíveis fatores de confusão. Questões relativas ao ambiente social, sobretudo em relação à violência percebida, devem ser consideradas na formulação de políticas públicas e ações de enfrentamento da insegurança alimentar.

## Abstract (en)

Food insecurity and urban violence are among the main public health problems in Brazil, with approximately half of the population having impaired access to food, in addition to most individuals having already experienced some violent event in their neighborhood. Studies have shown that violence in the neighborhood can be associated with food insecurity, however, in Brazil, this topic is little explored. This study aimed to verify the association between the perception of violence in the neighborhood and the risk of food insecurity. This study has a cross-sectional design, having been carried out with adults and older adults (n = 400) living in a health district of the city of Porto Alegre, Rio Grande do Sul State. A stratified sampling method was adopted. Data was collected by household interviews. The presence of risk of food insecurity and the perception of violence in the community were evaluated using instruments validated for the Brazilian population. Poisson regression with robust variance was used to estimate the crude and adjusted prevalence ratios (PR) and their respective 95% confidence intervals (95%CI). Individuals that perceived their neighborhood as violent were more likely to be at risk of food insecurity (PR = 1.35; 95%CI: 1.04-1.77). Perception of neighborhood violence is associated with risk of food insecurity, independently, after adjusting for potential confounders. Issues related to the social environment, especially in relation to perceived violence, must be considered in the creation of public policies and actions against food insecurity.

## Resumen

La inseguridad alimentaria y la violencia urbana se encuentran entre los principales problemas de salud pública en Brasil, con aproximadamente la mitad de la población con problemas de acceso a los alimentos, además de que una gran proporción de personas ya ha experimentado alguna situación de violencia en el barrio donde viven. Los estudios han demostrado que la violencia en el vecindario puede estar asociada con la inseguridad alimentaria; sin embargo, en Brasil este tema está poco explorado. El objetivo de este estudio fue verificar la asociación entre la percepción de violencia en el barrio y el riesgo de inseguridad alimentaria. Este estudio tiene un diseño transversal, y fue realizado con adultos y ancianos (n = 400) residentes en un área de salud de la ciudad de Porto Alegre, estado de Rio Grande do Sul. Se adoptó un método de muestreo estratificado. La recolección de datos se produjo mediante entrevistas domiciliarias. La presencia de riesgo de inseguridad alimentaria y la percepción de violencia en la comunidad se evaluaron mediante instrumentos validados para la población brasileña. Se utilizó la regresión de Poisson con varianza robusta para estimar la razón de prevalencia (RP) bruta y ajustada y sus respectivos intervalos de 95% de confianza (IC95%). Las personas que percibían que su vecindario era violento tenían más probabilidades de tener riesgo de inseguridad alimentaria (RP = 1,35; IC95%: 1,04-1,77). La percepción de violencia en el vecindario se asocia de forma independiente con el riesgo de inseguridad alimentaria, después de ajustar por posibles factores de confusión. Las cuestiones relacionadas con el entorno social, especialmente con respecto a la violencia percibida, deben considerarse al formular políticas y acciones públicas para combatir la inseguridad alimentaria.

## Introdução

A insegurança alimentar e a violência estão entre os principais problemas de saúde pública em todo o mundo ^1^. A insegurança alimentar se caracteriza pela falta de acesso regular a alimentos seguros e nutritivos para o crescimento e o desenvolvimento normais e uma vida ativa e saudável ^2^. Estimativas mostram que, no ano de 2022, 29,6% da população mundial estava em situação de insegurança alimentar moderada ou grave, não tendo, portanto, acesso à alimentação adequada ^1^. No Brasil, a insegurança alimentar atinge aproximadamente 125,2 milhões de pessoas ^3^. Já em relação à violência, estatísticas sobre homicídios colocam América Latina e Caribe como a região mais violenta do mundo, concentrando 27% dos homicídios, sendo o Brasil líder, em números absolutos, do ranking mundial com 10,4% de homicídios ^4^.

No que se refere à violência urbana no Brasil, dados de estudo realizado no ano de 2021 mostraram que em torno de 83% da população manifestou preocupação em ser vítima de um crime violento e 40% já haviam experimentado uma situação de violência nos dois anos que antecederam a pesquisa, além de se sentirem menos seguros e considerarem a violência como o maior risco que enfrentam em suas vidas diárias ^5^.

Apesar da violência urbana afetar de forma semelhante os indivíduos, alguns enfrentam maiores chances de serem vítimas de violência devido às desigualdades sociais, incluindo questões de raça/cor da pele, distribuição desigual de renda e local de moradia ^6^. Além disso, os recursos presentes em uma comunidade podem favorecer ou não o enfrentamento das situações de violência, principalmente entre populações vulnerabilizadas, como as que vivenciam a insegurança alimentar ^6^.

A Organização Mundial da Saúde (OMS) ^7^ define a violência como “*uso intencional da força física ou do poder real ou em ameaça, contra si próprio, contra outra pessoa, ou contra um grupo ou uma comunidade, que resulte ou tenha qualquer possibilidade de resultar em lesão, morte, dano psicológico, deficiência de desenvolvimento ou privação*”. A partir dessa definição, a OMS estabeleceu categorias nas quais a violência comunitária/urbana seria um tipo de violência interpessoal e que acontece entre pessoas que se conhecem ou não, ocorrendo principalmente fora de casa ^7^. Essa definição será utilizada neste estudo como marco teórico para abordar a violência na vizinhança. A violência na vizinhança autopercebida é um fator que se refere à percepção dos indivíduos diante dos aspectos sociais do ambiente da sua vizinhança, ou seja, da área ao redor do seu domicílio, na qual o indivíduo normalmente realiza atividades rotineiras ^8^.

Desde a década de 1990, vêm crescendo as pesquisas envolvendo as condições do ambiente da vizinhança e sua relação com oportunidades e barreiras para um estilo de vida mais saudável ^9^ ^,^ ^10^. A interação entre características individuais e fatores do ambiente pode moldar os resultados de saúde e a forma de viver dos indivíduos ^11^. Nesse sentido, estudos já demonstram que aspectos da vizinhança em que os indivíduos vivem e se relacionam, tais como a percepção de violência, podem estar associados à insegurança alimentar, favorecendo a sua prevalência ^9^ ^,^ ^10^.

As pesquisas em relação ao tema têm demonstrado que os indivíduos com insegurança alimentar apresentam maiores chances de vivenciar vários tipos de violência, tais como violência sexual, física e psicológica ^12^ ^,^ ^13^. Também se faz presente a violência na vizinhança, na qual observa-se que ser ameaçado, assediado e sentir-se inseguro ao caminhar pela vizinhança durante o dia se mostrou mais comum ^9^ ^,^ ^10^. Jackson et al. ^14^ verificaram que cerca de 23,4% das famílias estadunidenses com insegurança alimentar moderada e grave residiam em bairros violentos; em contrapartida, apenas 4% das famílias com alimentação suficiente residiam nessas áreas. Morar em bairros violentos aumentou de forma expressiva o risco de insegurança alimentar moderada e grave ^14^.

Apesar das altas taxas tanto de violência urbana quanto de insegurança alimentar, a relação entre esses fatores ainda é pouco explorada no Brasil. Dessa forma, o objetivo deste estudo é analisar associações da violência autopercebida e o risco de insegurança alimentar em indivíduos de um território de saúde da cidade de Porto Alegre, Estado do Rio Grande do Sul.

## Materiais e métodos

Este estudo utiliza dados da pesquisa *Estudo dos Determinantes Sociais e Ambientais da Alimentação e Nutrição: Uma Abordagem Ecossocial*, aprovada pelo Comitê de Ética em Pesquisa da Universidade Federal do Rio Grande do Sul (CAAE: 46934015.3.0000.5347). É uma pesquisa transversal de base populacional, realizada em um território de saúde na área central da cidade de Porto Alegre. A pesquisa foi realizada com pessoas adultas e/ou idosos de ambos os sexos.

### População do estudo

Aproximadamente 12 mil famílias vivem no território, caracterizado pela coexistência de áreas de alta e baixa vulnerabilidade. As áreas vulneráveis foram avaliadas por meio da localização geográfica predominantemente com ocupações irregulares, acesso prejudicado a serviços públicos essenciais, como serviço de esgoto sanitário e coleta de lixo. Além disso, foram considerados fatores como vias de circulação estreitas, ausência de calçadas ou de alinhamento irregular ^15^. Ainda foi utilizado um critério de renda *per capita* para a vulnerabilidade das áreas, sendo as áreas vulneráveis e não vulneráveis aquelas que apresentassem renda de R$ 1.700,00 e R$ 4.000,00, respectivamente ^16^.

### Tamanho da amostra

O tamanho da amostra foi calculado considerando os objetivos do estudo principal. Dessa forma, para esta análise, os cálculos do poder amostral para cada variável de exposição foram realizados *a posteriori*. Considerando os seguintes parâmetros: 95% de confiança e 80% de poder, a amostra final (n = 400) seria capaz de detectar uma razão de prevalência (RP) de aproximadamente 1,35 na percepção da violência quando associada ao desfecho.

### Amostragem

Foi adotado um método de amostragem estratificada. A amostra foi dividida em duas partes para garantir os diferentes estratos socioeconômicos pretendidos no estudo. Dessa forma, foram incluídas, aproximadamente, 250 famílias das áreas mais vulneráveis e a mesma proporção das áreas menos vulneráveis. Nas áreas mais vulneráveis, todos os participantes elegíveis das 250 famílias foram convidados a participar do estudo (amostragem censitária), incluindo 201 participantes (taxa de recusa de 16%). Nas áreas menos vulneráveis, utilizou-se amostragem aleatória para selecionar os domicílios, incluindo 199 participantes (taxa de recusa de 22%).

Apenas um indivíduo por domicílio foi incluído, quando havia mais de um adulto ou idoso, apenas um era escolhido de forma aleatória. A alternância entre os sexos dos participantes incluídos em cada domicílio foi realizada sempre que possível, ou seja, se uma mulher era incluída, tentava-se incluir um homem na próxima casa, de forma a manter a representatividade da amostra.

### Coleta de dados

Os dados foram coletados entre outubro de 2018 e junho de 2019 por equipe composta de nutricionistas e estudantes de nutrição previamente treinados. Um questionário padronizado, pré-codificado e previamente testado foi utilizado para obtenção dos dados.

Inicialmente, foi realizado o mapeamento das áreas, identificação dos domicílios e, posteriormente, feita a visita ao território pela equipe de pesquisa para identificar indivíduos que atendiam aos critérios de inclusão. Aqueles que aceitaram participar assinaram o termo de consentimento livre e esclarecido, sendo a coleta de dados realizada na visita inicial ao domicílio ou agendada para outro momento, preferencialmente no domicílio do entrevistado ou na unidade básica de saúde (UBS), quando solicitado pelo participante.

### Variável de desfecho

O desfecho do estudo foi o risco de insegurança alimentar, avaliado por meio da versão curta da *Escala Brasileira de Insegurança Alimentar* (EBIA) ^17^. A escala dicotômica “sim/não” identifica risco de insegurança alimentar quando o pesquisado responde afirmativamente a pelo menos um dos cinco itens, referentes ao acesso a alimentos nos últimos três meses: (1) você teve a preocupação de que a comida na sua casa acabasse antes que tivesse condição de comprar, receber ou produzir mais comida?; (2) a comida acabou antes que você tivesse dinheiro para comprar mais?; (3) você ficou sem dinheiro para ter uma alimentação saudável e variada?; (4) você ou algum adulto em sua casa diminuiu, alguma vez, a quantidade de alimentos nas refeições, ou pulou refeições, porque não havia dinheiro suficiente para comprar a comida?; (5) você alguma vez comeu menos do que achou que devia porque não havia dinheiro suficiente para comprar comida?

Apesar de a EBIA, em sua versão curta, apresentar como limitação a impossibilidade de avaliar o grau de insegurança alimentar, de distinguir entre domicílio com crianças e adolescentes, ou identificar causas/crenças que possam estar favorecendo a insuficiência alimentar, ela se mostrou adequada para ser utilizada na avaliação de risco de insegurança alimentar, possibilitando uma identificação ágil dos indivíduos mais vulneráveis para vivenciarem essa condição ^17^.

### Variável de exposição

A variável de exposição foi a medida autopercebida de violência na vizinhança. Para avaliar a percepção de violência, foi aplicado um questionário, validado e transculturalmente adaptado para a população brasileira por Santos et al. ^18^, que aborda características autorreferidas da vizinhança por meio de domínios.

A violência percebida na vizinhança foi avaliada por meio de cinco itens, referentes aos últimos seis meses: (1) com que frequência houve brigas que tenham envolvido o uso de armas na sua vizinhança?; (2) com que frequência houve uma discussão violenta entre vizinhos?; (3) com que frequência houve uma briga entre gangues (grupos ou facções rivais)?; (4) com que frequência houve violência sexual ou estupro?; e (5) com que frequência houve um roubo ou assalto? Os participantes responderam às perguntas por meio de uma escala de quatro pontos (1 = frequentemente, 2 = às vezes, 3 = raramente e 4 = nunca). Uma pontuação mais alta representa menor violência percebida.

O escore total da percepção do ambiente social foi obtido pela soma das respostas de cada participante; quanto maior o escore de percepção, menos violência na vizinhança foi percebida pelo indivíduo. Após, o escore foi convertido em quartis de tamanhos, aproximadamente, iguais.

### Covariáveis

Para a coleta de dados das covariáveis socioecômicas e demográficas, foi utilizado um questionário padronizado elaborado pelos pesquisadores contendo as seguintes variáveis: sexo (autodeclarado - feminino; masculino); idade (referida em anos completos); raça/cor da pele autodeclarada e categorizada conforme o censo de 2010 do Instituto Brasileiro de Geografia e Estatística (IBGE) ^19^ (branca; preta; parda; amarela; indígena); escolaridade (referida - não estudou; analfabeto; Ensino Fundamental completo ou incompleto; Ensino Médio completo ou incompleto; Ensino Superior completo ou incompleto; pós-graduação); estado civil (referida - solteiro; união estável; casado; viúvo; separado; divorciado); renda familiar mensal referida em faixas de salários mínimos (até 2; ≥ 3-5; > 5); vínculo empregatício (referido - sim; não); número de moradores no domicílio (referido - 1; 2-3; > 3); recebimento de benefícios (referido - não recebe; Bolsa Família; aposentadoria ou pensão; Benefício de Prestação Continuada - BPC; outros).

### Análises estatísticas

Os dados foram duplamente digitados no programa EpiData, versão 3.1 (http://www.epidata.dk/). As análises dos dados foram realizadas no software Stata 12.0 (https://www.stata.com) e SPSS, versão 18.0 (https://www.ibm.com/).

A normalidade da variável de exposição foi testada por meio de teste de Shapiro-Wilk. A análise do escore de percepção da vizinhança e a associação com as covariáveis foi apresentada por meio de mediana e intervalo interquartílico, e avaliadas por testes não paramétricos.

A presença de risco de insegurança alimentar e sua associação com as covariáveis foi descrita em frequência absoluta e relativa, utilizando-se o teste qui-quadrado de independência para a detecção de associações. A associação entre risco de insegurança alimentar e a variável de exposição foi explorada por meio de regressão de Poisson com variância robusta, estimando RP bruta e ajustada, e os respectivos intervalos de 95% de confiança (IC95%) ^20^.

As variáveis de ajuste na análise de regressão multivariável foram incluídas quando apresentaram associação (p < 0,20) com risco de insegurança alimentar e a exposição de interesse, observando-se a estrutura conceitual, conforme proposto por Victora et al. ^21^, que descrevem relações hierárquicas entre os fatores, sendo que os determinantes da insegurança alimentar sociodemográficos incluídos no nível mais distal e os determinantes econômicos no nível mais proximal.

A consistência interna da escala de percepção da violência foi estimada pelo coeficiente alfa de Cronbach padronizado. A consistência interna da EBIA foi estimada pelo coeficiente de Kuder-Richardson. O domínio que avaliou a violência autopercebida na vizinhança apresentou confiabilidade moderada (0,61), assim como a EBIA (0,68) ^22^.

## Resultados

Participaram do estudo um total de 400 indivíduos, predominantemente do sexo feminino (75%), com idade entre 19 e 49 anos (50,5%), de cor da pele branca (62,3%) e ensino médio completo (37%). Além disso, grande parte recebia entre 3 e 5 salários mínimos mensais (48,4%). A prevalência de risco de insegurança alimentar na população foi de 51,2%, sendo maior entre as mulheres e nos adultos jovens. Também, indivíduos com renda mais baixa, com famílias mais extensas e que viviam em áreas da vizinhança consideradas vulneráveis apresentaram maior percentual de risco de insegurança alimentar (Tabela 1).

**Tabela 1:** Prevalência de risco de insegurança alimentar e percepção de violência na vizinhança segundo características sociodemográficas e econômicas dos participantes. Porto Alegre, Rio Grande do Sul, Brasil (n = 400).

Variáveis	n (%)	Risco de insegurança alimentar [n (%)]	Valor de p	Percepção da violência (IIQ *)	Valor de p
Total	400 (100,0)	205 (51,2)			
Sexo			0,007 **^,^ ***	18 (16-19)	0,425 ^#^
Masculino	100 (25,0)	39 (39,0)		18 (16-19)	
Feminino	300 (75,0)	166 (55,3)		17 (15-19)	
Idade (anos)			< 0,001 **^, ##^		< 0,001**^, ###^
19-49	202 (50,5)	115 (56,9)		17 (15-18)	
50-59	110 (27,5)	66 (56,4)		18 (16-19)	
≥ 60	88 (22,0)	24 (27,3)		18 (17-20)	
Cor da pele			0,207 ***		0,436 ^#^
Branca	249 (62,3)	121 (48,6)		18 (16-19)	
Preta/Parda/Amarela	151 (37,8)	84 (55,6)		17 (15-19)	
Escolaridade [n = 395]			0,004 **^, ##^		0,137 ^###^
Ensino Fundamental incompleto	73 (18,3)	45 (37,7)		18 (16-20)	
Ensino Fundamental completo	73 (18,5)	40 (54,8)		18 (15-19)	
Ensino Médio completo	146 (37,0)	81 (75,4)		17 (16-18)	
Ensino Superior completo ou incompleto	103 (26,1)	38 (53,2)		18 (16-19)	
União estável			0,156 ***		0,561 ^#^
Sim	149 (37,3)	69 (46,3)		18 (16-19)	
Não	251 (62,7)	136 (54,2)		17 (15-19)	
Renda mensal (salários mínimos) [n = 399]			< 0,001 **^, ##^		0,877 ^###^
Até 2	134 (33,6)	96 (71,6)		17 (15-19)	
3-5	193 (48,4)	92 (47,7)		17 (16-19)	
> 5	72 (18,0)	17 (23,6)		18 (16-19)	
Benefício social			0,001 **^, ##^		0,305 ^###^
Não recebe	201 (50,2)	104 (51,7)		18 (15-19)	
Bolsa Família/BPC/Outros	52 (13,0)	38 (26,7)		17 (16-19)	
Aposentadoria/Pensão	147 (36,8)	63 (42,9)		18 (16-19)	
Vínculo empregatício			0,213 ***		0,012 **^, #^
Sim	245 (61,3)	119 (48,6)		17 (16-18)	
Não	155 (38,8)	86 (55,5)		18 (16-20)	
Área de moradia			< 0,001 **^,^ ***		0,075 ^#^
Vulnerável	201 (51,2)	125 (62,2)		17 (15-19)	
Não vulnerável	199 (49,8)	80 (40,2)		18 (16-19)	
Moradores no domicílio			< 0,001 **^, ##^		0,327 ^###^
1	66 (16,5)	25 (37,9)		18 (16-19)	
2-3	195 (48,8)	89 (45,6)		18 (15-19)	
> 3	139 (34,8)	91 (65,5)		17 (15-19)	
Risco de insegurança alimentar					0,001 ^#^
Sim				17 (15-18)	
Não				18 (16-19)	

BPC: Benefício de Prestação Continuada; IIQ: intervalo interquartil.

* Intervalo interquartil obtido a partir da pontuação do questionário de autopercepção da violência na vizinhança. A faixa de pontuação do questionário de autopercepção da violência pode variar entre 5 a 20 pontos. Quanto menor a pontuação, mais violência foi percebida na vizinhança, pelos entrevistados;

** Significância estatística considerada, p ≤ 0,05;

*** Teste de correção de continuidade de Yates;

^#^ Teste U de Mann-Whitney para amostras independentes;

^##^ Teste qui-quadrado de Pearson;

^###^ Teste de Kruskal-Wallis para amostras independentes.

A proporção de pessoas com percepção de menor violência na comunidade se mostrou associada aos indivíduos com maior idade e aos moradores sem vínculo empregatício. Indivíduos com risco de insegurança alimentar, quando comparados àqueles em segurança alimentar, perceberam mais violência na vizinhança em que vivem (Tabela 1).

Os resultados da associação entre o risco de insegurança alimentar e a percepção de violência ajustados para as covariáveis são apresentados na Tabela 2. Indivíduos com percepção de maior violência na vizinhança (Q1 e Q2) apresentaram maior risco de insegurança alimentar no modelo bruto e no modelo ajustado, confirmando que quanto maior a percepção de violência na comunidade, maior é a probabilidade de risco de insegurança alimentar (RP = 1,35; IC95%: 1,04-1,77).

**Tabela 2:** Razão de prevalência (RP) bruta e ajustada da associação entre o escore de violência percebida e risco de insegurança alimentar (n = 400).

Variáveis	Modelo bruto			Modelo ajustado	
	RP (IC95%)	Valor de p	RP (IC95%)	Valor de p
Escore de violência percebida (quartis)	n = 400	0,001 *	n = 395	0,008 *
Q1 (maior percepção)	1,48 (1,14-1,93)		1,35 (1,04-1,77)	
Q2	1,39 (1,06-1,83)		1,36 (1,04-1,78)	
Q3	1,10 (0,80-1,52)		1,10 (0,80-1,52)	
Q4 (menor percepção)	1,00		1,00	

IC95%: intervalo de 95% de confiança.

Nota: análise de regressão multivariável realizada por regressão de Poisson com variância robusta. Modelo bruto: efeito da violência autopercebida sem ajuste; Modelo ajustado: modelo ajustado para as variáveis idade e escolaridade. Nenhuma variável socioeconômica (nível proximal da estrutura conceitual) atendeu ao critério de ajuste estabelecido (associação com o desfecho e com a exposição no nível de significância menor que 20%, p < 0,20), cabendo, portanto, a construção de somente um modelo ajustado que incluiu as variáveis do nível distal da estrutura conceitual que atenderam ao critério de ajuste (idade e escolaridade).

* Significância estatística considerada, p ≤ 0,05.

## Discussão

Os resultados deste estudo mostram que a prevalência de risco de insegurança alimentar é elevada e que indivíduos com percepção de maior violência na vizinhança tiveram maior probabilidade de apresentar o desfecho.

A prevalência de risco de insegurança alimentar na amostra estudada foi de 51,2%, e apesar de o instrumento utilizado neste estudo não determinar os níveis de insegurança alimentar, os resultados permitem inferir que essa prevalência foi maior do que a encontrada na *Pesquisa de Orçamentos Familiares* (POF) de 2017-2018 (36,7%) ^23^ e próxima ao encontrado no *II Inquérito de Insegurança Alimentar no Contexto da Pandemia da COVID-19*, cujas prevalências foram de 58,7% de insegurança alimentar no Brasil e 47,6% no Estado do Rio Grande do Sul em 2022 ^3^ ^,^ ^24^.

De forma similar ao encontrado em outros estudos, a maior prevalência de risco de insegurança alimentar em mulheres, nos mais jovens, naqueles com menor renda e escolaridade, e nas famílias mais extensas comprova que os fatores sociodemográficos e econômicos se fazem presentes como determinantes na insegurança alimentar em diversos países ao redor do mundo ^9^ ^,^ ^24^ ^,^ ^25^. O Brasil é um país com altas taxas de desigualdades sociais com distribuição assimétrica de recursos e oportunidades, limitando o direito à alimentação adequada e saudável em diferentes grupos da sociedade. Estima-se que seis em cada dez domicílios cujo responsável seja mulher vivenciam algum grau de insegurança alimentar no Brasil ^3^. As chances de indivíduos ou grupos populacionais vivenciarem insegurança alimentar se reduzem quando há acréscimo de recursos monetários, sendo a escolaridade um fator que possibilita a elevação da renda média dos indivíduos, enquanto a presença de mais moradores no domicílio pode comprometer uma parcela maior dos recursos familiares ^26^.

Embora a renda, a pobreza, as composições familiares, as desigualdades de gênero e os baixos índices de escolaridade sejam importantes determinantes sociais e econômicos da insegurança alimentar, há aspectos da vizinhança que podem influenciar nas condições para um ambiente com maior ou menor insegurança alimentar. Nesse sentido, o ambiente social na vizinhança poderá resultar em uma percepção mais segura ou mais violenta da vizinhança. Nossos resultados demonstraram que indivíduos com uma percepção de maior violência em sua vizinhança apresentavam maior probabilidade de risco de insegurança alimentar. Esses dados vão ao encontro de outros estudos, como: o realizado por Jackson et al. ^14^ nos Estados Unidos, que demonstrou que viver em bairros violentos aumentou o risco de insegurança alimentar moderada e grave em 283%; o realizado por Mmari et al. ^10^ com jovens em Baltimore (Estados Unidos), que demonstrou que pessoas com a percepção de viverem em vizinhanças inseguras apresentaram 3,68 vezes mais chances de vivenciarem insegurança alimentar; e o realizado por Ogbu et al. ^27^ com adultos nigerianos, o qual identificou que indivíduos residentes em bairros considerados inseguros apresentaram maior prevalência de insegurança alimentar moderada e grave, em comparação aos indivíduos que consideraram seu bairro seguro.

Além disso, os estudos têm demonstrado também que indivíduos com insegurança alimentar vivem em comunidades com altos níveis de conflitos sociais e violência ^28^ ^,^ ^29^ ^,^ ^30^ ^,^ ^31^. Mmari et al. ^10^ identificaram que indivíduos em insegurança alimentar tinham mais frequentemente a percepção que seus bairros eram violentos, confiando menos na comunidade em que viviam. Maguire-Jack et al. ^32^ demonstraram que a pobreza no bairro onde os indivíduos viviam estava relacionada a agressões físicas e psicológicas. Mulheres em insegurança alimentar obtiveram níveis mais baixos de segurança percebida na vizinhança do que aquelas em segurança alimentar. Ser ameaçado e assediado e sentir-se inseguro ao caminhar pela vizinhança durante o dia também foi mais comum entre aqueles que apresentavam insegurança alimentar ^9^ ^,^ ^10^.

Estudo realizado em Atlanta (Estados Unidos) ^33^ com o objetivo de compreender a relação entre baixo acesso aos alimentos e ferimento por arma de fogo demonstrou que o baixo acesso aos alimentos foi associado à incidência de ferimento por arma de fogo. Os pontos com insegurança alimentar na cidade se sobrepuseram aos que apresentavam maiores níveis de violência. A insegurança alimentar moderada e a grave foram as que obtiveram maior incidência de vítimas, demonstrando que esses indivíduos estão expostos a ambientes menos seguros. A relação entre violência na vizinhança e insegurança alimentar parece demonstrar uma relação bidirecional. Ao mesmo tempo em que indivíduos que vivem em ambientes com violência apresentam maior probabilidade de estarem em risco de insegurança alimentar, estar em situação de insegurança alimentar é uma condição de vulnerabilidade que limita a possibilidade dessas pessoas buscarem por moradias em ambientes mais seguros e com recursos físicos e sociais adequados. Assim, tais indivíduos acabam por permanecer em bairros desfavorecidos, marginalizados, violentos, com poucas oportunidades de emprego, altos níveis de pobreza e degradação física ^34^. Estudos sugerem que a associação observada entre violência na comunidade e insegurança alimentar pode também ocorrer por meio da saúde mental prejudicada entre esses indivíduos. Pessoas que vivem em bairros violentos ou que consideram com baixa segurança, apresentam mais desfechos em saúde mental, como ansiedade, estresse e depressão, podendo ser resultado das condições de vida e das desigualdades enfrentadas por eles ^35^. A própria presença de insegurança alimentar já foi associada a diversos desfechos negativos de saúde mental, sugerindo que níveis mais intensos de insegurança alimentar estejam relacionados, mesmo que parcialmente, a esses aspectos. Assim, pode haver perda no potencial produtivo desses indivíduos e prejuízo na busca por equipamentos públicos que possam favorecer o acesso a recursos de saúde e alimentação, como os benefícios sociais ^10^ ^,^ ^14^.

Além disso, as experiências individuais sobre a percepção de violência na vizinhança podem ser responsáveis também pelo distanciamento entre os vizinhos e a baixa participação comunitária, que pode ocorrer como resposta ao medo de ser vítima de violência, por exemplo. Estudo realizado com adultos de Chicago (Estados Unidos) ^36^ identificou que a violência na comunidade foi capaz de diminuir em 3,3 pontos a interação dos indivíduos com a rede social, ou seja, diminuindo a coesão social no bairro. Swart et al. ^37^ identificaram, em Joanesburgo, na África do Sul, região de alta vulnerabilidade social, que o medo de ser vítima de um crime violento na sua vizinhança foi capaz de impedir suas atividades rotineiras, como: utilizar de transportes públicos; caminhar até as lojas; caminhar para o trabalho/cidade; ir a parques; permitir que as crianças brinquem livremente. O enfraquecimento das relações entre os indivíduos a partir da violência comunitária pode impedir ações coletivas de enfrentamento e o acesso a redes de apoio, impactando na probabilidade de vivenciar insegurança alimentar, principalmente em comunidades pobres e marginalizadas ^36^.

Em nosso estudo, também identificamos uma diferença geracional na percepção de violência na vizinhança. Indivíduos com mais idade tiveram uma percepção de menor violência em sua comunidade, quando comparados aos mais jovens. Isso poderia ocorrer devido aos indivíduos de mais idade apresentarem crenças em relação à aceitabilidade da violência no espaço onde vivem ou até mesmo perceberem as evoluções e melhorias, mesmo que pequenas, que a vizinhança apresentou ao passar do tempo. Estudo qualitativo realizado em uma área altamente vulnerável de Nova Jersey (Estados Unidos) ^38^ identificou, por meio de dois grupos focais, que participantes de maior idade conseguiam identificar melhorias nos seus bairros tendo uma percepção mais positiva em relação à segurança/violência no ambiente em que viviam. Em contrapartida, residentes mais jovens mantiveram uma percepção relativa às mudanças, ou seja, consideraram sua vizinhança, apesar das alterações, violenta. Estudo realizado com mulheres de baixa renda da Filadélfia (Estados Unidos) ^39^ também identificou que as mulheres mais velhas tiveram menor preocupação em relação a crimes e à segurança na sua vizinhança, relatando menores níveis de desordens, tanto físicas quanto sociais, no bairro. Indivíduos com mais idade podem apresentar vivências diversas e, consequentemente, padrões diferentes para avaliar a comunidade em que vivem. Apesar dos estudos sobre o tema, as evidências sobre essas relações ainda são controversas, uma vez que outras pesquisas demonstram que os indivíduos mais jovens tendem a ter suas decisões menos afetadas pela violência no bairro, apresentando uma percepção de menor violência na comunidade. Han et al. ^40^ realizaram estudo em bairros de baixa renda de Los Angeles (Estados Unidos) e identificaram que adultos e idosos eram significativamente mais propensos a deixarem de visitar parques no bairro em que viviam, por exemplo, após um crime violento, entretanto isso não se mostrou semelhante quando analisados indivíduos mais jovens.

Os resultados encontrados neste estudo demonstram a necessidade de políticas públicas que possam agir sobre os determinantes sociais, principalmente por meio de abordagens nos territórios/vizinhanças em que vivem. Diante desse processo, a atenção primária à saúde (APS) com a Estratégia Saúde da Família (ESF) se destaca nas abordagens de saúde territorializadas, ofertando ações de saúde individuais, familiares e coletivas, prestadas por equipe multiprofissional, considerando o território como mutável e transformador das realidades sociais ^41^. A saúde mental prejudicada dos indivíduos que vivem em ambientes violentos pode ser um fator que favorece o maior risco de insegurança alimentar. Nesse contexto, as equipes mínimas da ESF devem possuir apoio de equipes ampliadas com profissionais da área da psicologia, capazes de qualificar a assistência ofertada à população, tendo como experiência a atuação das equipes no Núcleo de Apoio a Saúde da Família (NASF) e da atenção básica, ou, mais recentemente, a formação das equipes multiprofissionais na APS (eMulti) ^42^.

É necessário fortalecer ações que dialoguem com a Política Nacional de Promoção da Saúde, que estabelece o fomento ao planejamento de ações territorializadas de promoção da saúde com a construção de ambientes e territórios saudáveis no espaço de vida e trabalho dos indivíduos. Considerar a diminuição dos níveis de violência urbana, incentivo à cultura da paz e dos direitos humanos, como ações prioritárias, de modo a criar oportunidades de convivência, solidariedade entre os indivíduos e a redução da violência no ambiente em que se vive ^43^.

Ainda, são essenciais estratégias que visem à diminuição da insegurança alimentar como um fator determinante para a saúde e bem estar da população. Nesse aspecto, foi lançado, em 2024, o Plano Brasil sem Fome, que dispõe de 80 ações e programas intersetoriais, envolvendo os 24 Ministérios que compõem a Câmara Interministerial de Segurança Alimentar e Nutricional (CAISAN). O fortalecimento do Sistema Nacional de Segurança Alimentar e Nutricional (SISAN), o acesso à renda e a redução da pobreza, o desenvolvimento de uma política de agroecologia capaz de estimular a agricultura familiar e o avanço dos equipamentos públicos para a segurança alimentar são eixos estratégicos ^44^, que, em conjunto com outras iniciativas que busquem diminuir as desigualdades sociais, podem impactar de forma importante o acesso ao direito fundamental à alimentação adequada e saudável.

### Limitações do estudo

Este estudo apresentou algumas limitações. A amostra não é representativa da população em geral, o que limita a generalização dos achados. Embora tenham sido empregados esforços para atenuar o viés de seleção da amostra, o sexo feminino foi representado em maior número na amostra final. As medidas de exposição foram obtidas a partir da percepção do entrevistado, o que pode apresentar distorções a partir das vivências individuais, diferindo da realidade na vizinhança. Por ser um estudo transversal, os caminhos causais não podem ser determinados. Além disso, a EBIA na sua versão curta não permite identificar os níveis de insegurança alimentar no domicílio, sendo utilizada, neste estudo, exclusivamente como uma forma de avaliação do risco de insegurança alimentar.

## Conclusão

Os resultados deste estudo reforçam a necessidade de ampliar o olhar sobre os determinantes da insegurança alimentar, tanto nas investigações como na elaboração de políticas e programas que visem o seu enfrentamento, tendo em vista sua diversidade e sua complexidade. Assim, é essencial compreender os processos comunitários, as características sociais e econômicas, bem como os contextos de vida aos quais as pessoas pertencem. A partir dos resultados da pesquisa e considerando a necessidade de ações com enfoques intersetoriais e interprofissionais, é possível reforçar o papel das equipes de atenção básica à saúde, as quais conhecem a organização de vida e atuam em abordagens focadas no território, enfrentando cotidianamente a problemática da violência urbana e da insegurança alimentar que atinge parcela significativa da população.
